# Adrenal Insufficiency in Metastatic Lung Cancer

**DOI:** 10.14740/wjon890w

**Published:** 2015-06-12

**Authors:** Filipe Carvalho, Fernanda Louro, Raed Zakout

**Affiliations:** a Physical Medicine and Rehabilitation Department, Hospital Prof. Doutor Fernando Fonseca, E.P.E., Portugal; b Department of Medicine I, Hospital Prof. Doutor Fernando Fonseca, E.P.E., Portugal

**Keywords:** Lung cancer, Metastasis, Adrenal insufficiency

## Abstract

We report a case of adrenal insufficiency in patient with lung cancer. Although adrenal metastases are common in cancer patients, adrenal insufficiency is a rare occurrence. Diagnosis and treatment of adrenal insufficiency will improve the physical status and the quality of life in those patients.

## Introduction

Adrenal glands are a common site for metastases from solid tumors [1]. However, the occurrence of adrenal insufficiency is a very uncommon event [2], as 90% of the glands must be destroyed to cause adrenal insufficiency [3]. We present a case of a patient with a previously diagnosed non-small cell lung cancer (NSCLC) with known metastatic involvement of the adrenals, which evolved into adrenal failure.

## Case Report

A 60-year-old male patient was admitted to the hospital with several-week history of weakness, adynamia and progressive loss of deambulation, having become bedridden at the last days before the hospitalization.

He was diagnosed 7 months ago with NSCLC in stage IV, with metastasis to the contralateral lung and both adrenal glands. He also had a history of primary hypertension.

On admission, he was dehydrated and his blood pressure (BP) was 77/45 mm Hg. The rest of the physical examination was irrelevant. Laboratory analyses revealed Hb 12.8 g/dL, sodium 117 mmol/L, potassium 6.2 mmol/L, creatinine 3.09 mg/dL, blood urea nitrogen (BUN) 123 mg/dL, and C-reactive protein 5.16 mg/dL. Arterial blood gases sample (ambient air) showed pH 7.437, PCO_2_ 19 mm Hg, PO_2_ 98 mm Hg, HCO^3-^ 12.6 mmol/L, and SO^2-^ 98%.

He initiated normal saline infusion and cation exchange resin. Despite sorotherapy he remained hypotensive (BP 70/40 mm Hg), but had mild improvement on laboratory evaluation as follows: sodium 127 mmol/L, potassium 5.3 mmol/L, creatinine 1.85 mg/dL and BUN 48 mg/dL.

To assess the adrenal function, the next analyses were realized: serum cortisol 3.48 μg/mL (normal 4.3 - 22.4), serum ACTH 181.8 pg/mL (4.7 - 48.8), serum aldosterone < 1 ng/dL (1 - 16), plasma renin activity 31.76 μg/mL/h (0.37 - 3.84) and 24-h urinary cortisol 6.16 μg (28.5 - 213.7). Computed tomography (CT) scan of the abdomen showed a large nodular enlargement of both adrenal glands.

The diagnosis of metastatic primary adrenal insufficiency was established, and he initiated hormonal replacement therapy with hydrocortisone 5 mg tid and fludrocortisone 0.05 mg qd.

In the following days, the patient presented rapid clinical improvement, with normalization of his blood pressure. Laboratory evaluation also showed normal electrolytes and renal function: Hb 9.06 g/dL, sodium 137 mmol/L, potassium 3.9 mmol/L, creatinine 1.17 mg/dL, and BUN 21 mg/dL.

On discharge the patient had BP 118/64 mm Hg, and in the subsequent follow-ups he had recovered his autonomous deambulation.

## Discussion

Metastases to the adrenal glands are a frequent finding in patients with advanced malignancies like lung, breast, gastric, and colorectal cancer, melanoma, and non-Hodgkin’s lymphoma. This occurs likely due to their rich sinusoidal supply [4]. For instance, large autopsy series have shown that the prevalence of adrenal metastases was as much as 42% in patients with lung cancer [5].

Most data on patients with adrenal insufficiency due to adrenal metastases have been reported as clinical case reports [3, 4, 6-27]. In some of these cases adrenal insufficiency was the presenting feature of occult malignancy [4, 6-8, 10, 12, 23-25, 27]. In a prospective study, one-third of patients with bilateral adrenal involvement by metastatic cancer had adrenal insufficiency based on cosyntropin test [28]. Seidenwurm et al found four cases of adrenal insufficiency in 21 patients (19%) with bilateral adrenal metastases or unilateral adrenal metastases in patients with contralateral adrenalectomy [3]. On the other hand, in one retrospective study of 30 years with 464 patients with adrenal metastatic disease from various tumors, only five of these patients developed adrenal insufficiency [2]. The differences in the frequency of adrenal insufficiency in these studies seem mainly to be due to the pre-selection of the patients and variable diagnostic criteria of adrenal insufficiency, ranging from solely clinical to stimulation tests, with some interpretations of test results questionable due to paraneoplastic endogenous stimulation of the adrenal cortex and the consequent blunt response of cortisol to those tests [29].

Majority of patients with adrenal metastases have activation of the hypothalamic-pituitary-adrenal axis with strongly elevated cortisol levels [29, 30]. This is explained by the activated hypothalamic-pituitary-adrenal axis due to increased physical and mental stress of tumor patients [30, 31] and interaction between the tumor, the immune and the endocrine system [32]. This could explain why adrenal insufficiency is infrequent and develops only in patients with large bilateral metastases [29], with destruction of more than 90% of the functional cortex [33].

In addition to adrenal insufficiency that develops owing to replacement of both glands by metastases, other causes of adrenal insufficiency in cancer patients include hemorrhagic necrosis of adrenals in the context of metastatic infiltration, impaired adrenal synthesis in patients being treated with some anti-cancer drugs, metastases to the pituitary gland causing secondary adrenal insufficiency [27], and finally, even patients whose adrenal dysfunction is asymptomatic may have inadequate adrenal reserve if they become seriously ill, or can develop relative adrenal insufficiency after withdrawal of steroids included in many treatment protocols. On the other hand, steroids included in many treatment protocols may mask the presence of preexistent adrenal insufficiency.

In patients with cancer, adrenal insufficiency may go unrecognized because the symptoms, such as weakness, anorexia, nausea, vomiting, dehydration and orthostatic hypotension, and laboratory abnormalities, such as hyponatremia, hyperkalemia, and pre-renal azotemia are nonspecific and may be attributed to the underlying progressive malignancy or to the cancer therapy. On the other hand, terminal stage cancer patients can have symptoms that overlap with symptoms of adrenal insufficiency. For instance, in a prospective study of seven patients with adrenal metastases, three of them had symptoms suggestive of adrenal insufficiency that was not confirmed by cosyntropin stimulation test [34].

Patients with bilateral adrenal metastases, particularly those with large bilateral metastases, should be evaluated for the presence of adrenal insufficiency and receive careful follow-up, even if the baseline testing is normal. The treatment of the disease involves life-long administration of glucocorticoids and mineralocorticoids, which will improve the quality of life.
